# Activity screening of environmental metagenomic libraries reveals novel carboxylesterase families

**DOI:** 10.1038/srep44103

**Published:** 2017-03-08

**Authors:** Ana Popovic, Tran Hai, Anatoly Tchigvintsev, Mahbod Hajighasemi, Boguslaw Nocek, Anna N. Khusnutdinova, Greg Brown, Julia Glinos, Robert Flick, Tatiana Skarina, Tatyana N. Chernikova, Veronica Yim, Thomas Brüls, Denis Le Paslier, Michail M. Yakimov, Andrzej Joachimiak, Manuel Ferrer, Olga V. Golyshina, Alexei Savchenko, Peter N. Golyshin, Alexander F. Yakunin

**Affiliations:** 1 Department of Chemical Engineering and Applied Chemistry, University of Toronto, Toronto, ON, M5S 3E5, Canada; 2 School of Biological Sciences, Bangor University, Gwynedd LL57 2UW, UK; 3 Midwest Center for Structural Genomics and Structural Biology Center, Biosciences Division, Argonne National Laboratory, Argonne, Illinois 60439, USA; 4 Commissariat à l’Energie Atomique et aux Energies Alternatives (CEA), Direction de la Recherche Fondamentale, Institut de Génomique, Université de d’Evry Val d’Essonne (UEVE), Centre National de la Recherche Scientifique (CNRS), UMR8030, Génomique métabolique, Evry, France; 5 Université de d’Evry Val d’Essonne (UEVE), Centre National de la Recherche, Scientifique (CNRS), UMR8030, Génomique métabolique, Commissariat à l’Energie, Atomique et aux Energies Alternatives (CEA), Direction de la Recherche, Fondamentale, Institut de Génomique, Evry, France; 6 Institute for Coastal Marine Environment, CNR, 98122 Messina, Italy; 7 Institute of Catalysis, CSIC, Madrid 28049, Spain

## Abstract

Metagenomics has made accessible an enormous reserve of global biochemical diversity. To tap into this vast resource of novel enzymes, we have screened over one million clones from metagenome DNA libraries derived from sixteen different environments for carboxylesterase activity and identified 714 positive hits. We have validated the esterase activity of 80 selected genes, which belong to 17 different protein families including unknown and cyclase-like proteins. Three metagenomic enzymes exhibited lipase activity, and seven proteins showed polyester depolymerization activity against polylactic acid and polycaprolactone. Detailed biochemical characterization of four new enzymes revealed their substrate preference, whereas their catalytic residues were identified using site-directed mutagenesis. The crystal structure of the metal-ion dependent esterase MGS0169 from the amidohydrolase superfamily revealed a novel active site with a bound unknown ligand. Thus, activity-centered metagenomics has revealed diverse enzymes and novel families of microbial carboxylesterases, whose activity could not have been predicted using bioinformatics tools.

We are living on a “Planet of microbes” with microorganisms and their communities occupying every biological niche and representing the largest part of the global biodiversity. Our present knowledge of microorganisms and their enzymes is based largely on laboratory studies of pure microbial cultures. However, more than 99% of environmental microbes cannot be cultivated in the lab using routine techniques and therefore cannot be studied using classical experimental approaches^1^,^2^,^3^. Metagenomics has emerged as a strategic approach to explore unculturable microbes through the sequencing and analysis of DNA extracted from environmental samples, as well as using such experimental methods as DNA hybridization, gene expression, proteomics, metabolomics, and enzymatic screening^4^,^5^,^6^,^7^. The remarkable contribution of this approach to global DNA sequencing efforts has been demonstrated by several large scale metagenomic projects including the Sargasso Sea sampling (over one million novel protein encoding genes), the Global Ocean Survey (over six million genes), and human gut microbiome studies (over 3 million genes)^8^,^9^,^10^,^11^.

Due to the progress in DNA sequencing technology, the number of sequenced genomes and protein sequences in public databases has expanded exponentially. As of July 2015, the UniProtKB/TrEMBL database contained over 50 million sequences (European Bioinfomatics Institute (EBI) website http://www.ebi.ac.uk/). However, it is estimated that the global protein universe of microorganisms exceeds 10^12^ proteins indicating that we know astonishingly little about microbial proteins and enzymes^12^,^13^. Even more, based on conservative estimates, over 50% of the sequences available in the databases have uncertain (general), unknown, or incorrectly annotated functions^14^. Therefore, the direct experimental determination of protein function or enzyme activity for millions of biochemically uncharacterized proteins or genes of unknown function represents one of the major challenges in postgenomic biology. In addition to sequence similarity-based and comparative genomics methods of gene function prediction, there are several experimental approaches to annotation including analysis of gene or protein interactions, gene expression, gene knockouts, protein localization, and protein structures^15^,^16^,^17^,^18^,^19^. However, in most cases, these approaches produce predictions or general annotations of biochemical or cellular function requiring subsequent experimental verification. In contrast, screening of purified proteins or metagenome gene libraries for enzymatic activity represents a direct experimental approach to identify the biochemical function of unknown proteins^5^,^7^,^20^,^21^,^22^. The feasibility and merits of general and specific enzymatic assays for screening of purified proteins and metagenome libraries has already been demonstrated for many hydrolases and oxidoreductases, two very broad classes of enzymes^5^,^7^,^20^,^21^,^23^.

The metagenomic enzyme screening approach involves directly assaying proteins expressed from environmental DNA in a surrogate host (most often *E. coli*) for enzymatic activity against a specific chemical substrate^24^. An alternate approach is to clone environmental DNA fragments into a lambda phage-based system and to screen for enzymatic activities directly on phage plaques^25^. Enzymatic screening of metagenome libraries provides the possibility to mine for new enzyme activities and discover novel families of enzymes with no sequence similarity to previously characterized proteins. This method has greatly expanded the number of novel enzymes, including over 130 new nitrilases and many cellulases, carboxylesterases, and laccases^26^,^27^,^28^. A recent high-throughput metagenomics project has identified over 27,000 putative carbohydrate-active genes in the cow rumen metagenome and demonstrated the presence of glycosyl hydrolase activity in 51 out of 90 tested proteins^29^. In addition, metagenomes from several extreme environments have revealed a rich biochemical diversity of enzymes adapted to function under extreme conditions, such as low/high temperatures, low/high pH, and high salt concentrations or high pressure^5^,^30^,^31^. Biochemical and structural characterization of these enzymes has revealed different molecular mechanisms of adaptation to extreme environmental conditions^32^,^33^,^34^. A recent analysis of metagenome screening works published in the last two decades revealed that these studies identified almost 6,000 genes with 70% of them representing carboxylesterases and lipases^35^. Based on sequence, most known carboxylesterases and lipases belong to the large protein superfamilies of α/β hydrolases and β-lactamases and have been classified into 16 families^36^,^37^,^38^. Since these enzymes are of high interest for applications in biotechnology, a significant number of these proteins have been characterized both structurally and biochemically, mostly esterases from the α/β hydrolase superfamily^36^,^39^,^40^,^41^.

Here we present the results of enzymatic screening of 16 metagenomic gene libraries from different environments for novel carboxylesterases. We have identified over 700 positive clones, from which 80 selected genes were expressed in *E. coli*, and their esterase activities were confirmed using additional assays. Four enzymes representing unknown (DUF3089) and hypothetical (MGS0084) proteins, cyclase-like enzymes (PF04199), as well as polyester hydrolyzing and lipolytic enzymes were characterized biochemically including substrate and temperature profiles. The active site residues of new enzymes were identified using site-directed mutagenesis, and the crystal structure of a metal-dependent cyclase-like esterase provided insight into the molecular mechanisms of its activity.

## Results and Discussion

### Enzymatic screening of metagenome libraries for carboxylesterase activity

**Figure 1:**
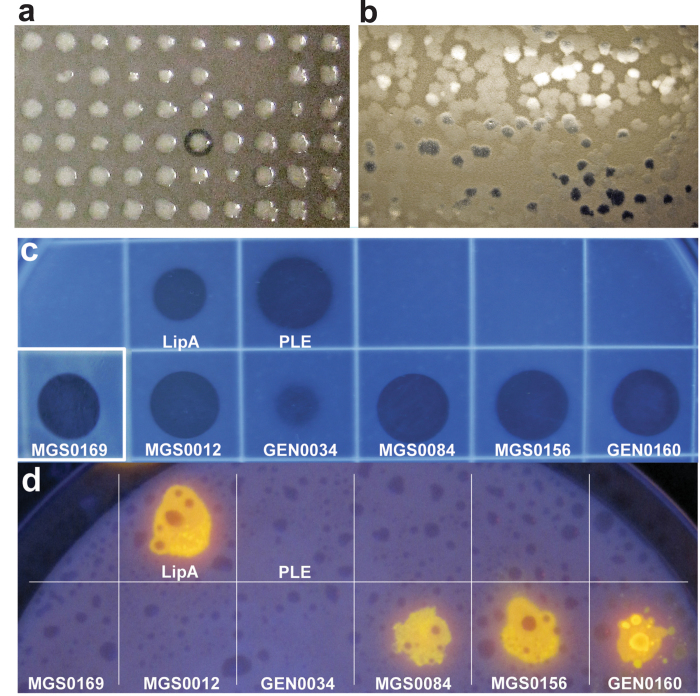
Agar-based screening of metagenomic libraries and purified metagenomic proteins for esterase and lipase activities. (**a**) Fosmid library screening (tributyrin plate); (**b**) lambda-Zap library screening (tributyrin plate); (**c**) screening of purified proteins (activity validation; tributyrin plate); (**d**) screening of purified proteins for lipase activity (olive oil plate). Purified proteins (50 μg, except for MGS0169 and GEN0034, for which 100 μg were used) were spotted onto agar plates containing 1% tributyrin (**a**,**b**,**c**) or 3% olive oil (**d**) and incubated overnight at 30 °C. Commercial enzymes pig liver esterase (PLE, 5 μg) and Lipase A (LipA, 5 μg) were used as positive controls. A clearing on tributyrin plates indicates esterase activity while fluorescence visualized under UV light (bright yellow spots) on olive oil plates indicates lipase activity.

To probe the biochemical diversity of carboxylesterases from uncultured microbes of environmental metagenomes, we screened 16 metagenome DNA libraries prepared from different geographic sites including various marine environments, soils, and waste treatment facilities (Table 1, Supplementary Table S1). The environments include moderate to hypersaline (3.8% to 10% NaCl, w/vol) conditions, low to elevated temperatures (3 °C to 50 °C), as well as sites contaminated with petroleum or heavy metals, from public or industrial wastewater sludge digesters (Supplementary Table S1). Overall, we screened over 1 million fosmid and Lambda-ZAP clones (approximately 7,000 Mbp DNA) for the ability to degrade tributyrin, generating a total 714 positive fosmid and Lambda-ZAP clones (Fig. 1, Table 1). Lambda-ZAP clones (208) and 178 fosmids (from the total 506) were sequenced by primer walking or using Illumina HiSeq, respectively. All genes predicted to have hydrolytic enzyme activity were cloned for protein purification. Where no sequence similarity to known esterases was found (two Haven library clones), we subcloned all predicted open reading frames and identified the presence of esterase activity in one hypothetical protein (MGS0084) and one predicted cyclase (MGS0169).

We confirmed the presence of esterase activity in 80 selected genes using agar plates with 1% tributyrin (Fig. 1, Supplementary Table S2). These enzymes were also tested for the presence of lipase activity, based on the ability to hydrolyze long chain-length lipids (C16, C18), using an olive oil agar plate assay (Fig. 1d). Three enzymes (MGS0084, MGS0156 and GEN0160) out of 80 tested clones were found to have lipase activity (Fig. 1), consistent with previous metagenome screens where low frequency of lipase activity was reported^7^.

### Sequence analysis and enzyme families of identified metagenomic esterases

**Figure 2:**
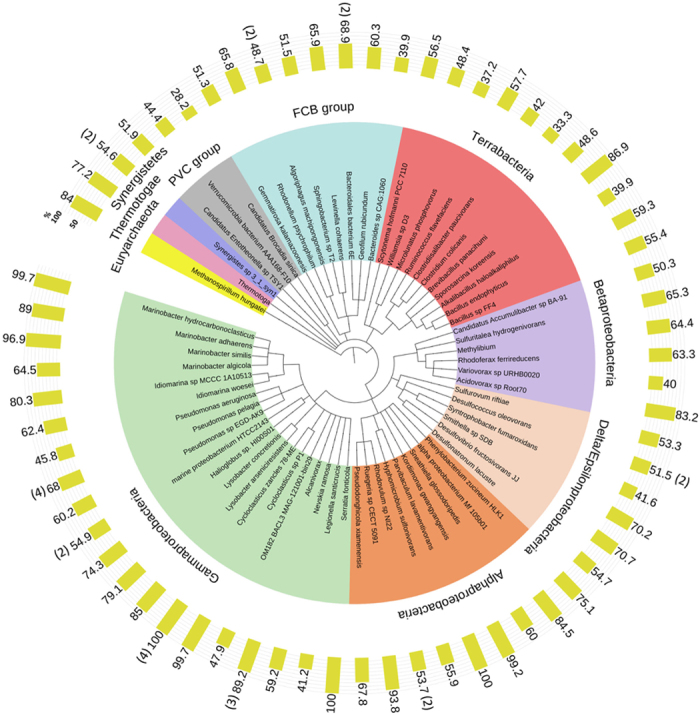
Phylogenetic distribution of 80 metagenomic carboxylesterases and their top sequence homologues across the tree of life. The numbers on yellow bars show sequence identity (%) of metagenomic esterases to protein sequences from indicated organisms. For organisms/genera with more than one metagenome esterase homologue, the means are indicated (with the number of esterases shown in brackets).

BLASTp searches of the NCBI database using 80 validated metagenomic esterases as queries indicated that most of these proteins represent genuine metagenomic enzymes with just 11 sequences from known genomes including *Alcanivorax borkumensis, Cycloclasticus* sp. 78-ME, *Marinobacter hydrocarbonoclasticus, Parvibaculum lavamentivorans,* and *Serratia fonticola* (99–100% identity) (Fig. 2, Supplementary Table S2). Sixty-nine remaining esterases showed 28–98% sequence identity to sequences from the NCBI database with most sequences within the range of 50–80% identity. Analysis of phylogenetic distribution of the 80 validated metagenomic esterases revealed that these proteins and their top sequence homologues are present in a broad range of Gram-positive and Gram-negative microorganisms with most proteins found in Proteobacteria (52 proteins), Terrabacteria (11 proteins), and the Fibrobacteres, Chlorobi and Bacteroidetes (FCB) group (10 proteins) (Fig. 2).

**Figure 3:**
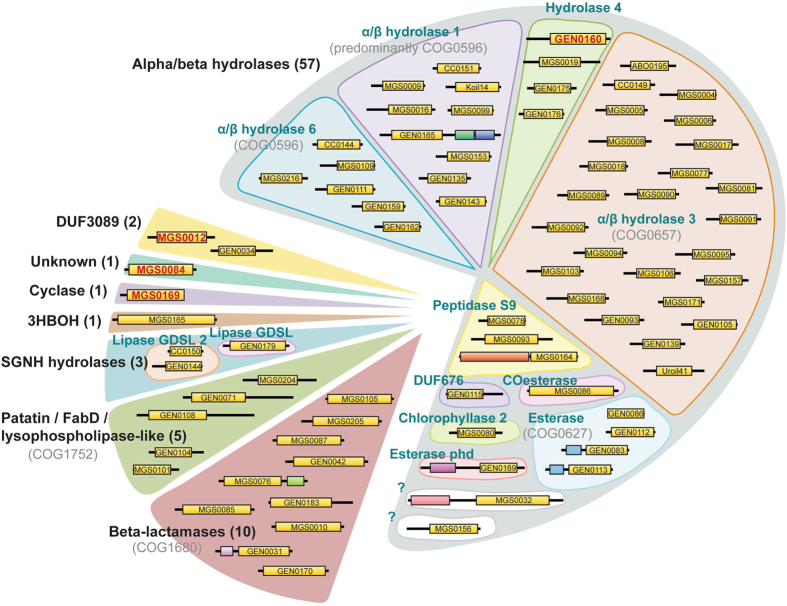
Protein family classification of 80 carboxylesterases identified by activity screening of metagenomic libraries. For all genes, the presence of esterase activity was confirmed by individual subcloning and expression in *E. coli*. Enzymes were classified into protein families using HMMER and BLAST searches of Pfam and COG databases. Polypeptide chain lengths are proportional to size and predicted domains are depicted as boxes. Domains predicted/confirmed to confer esterase activity are shown in yellow. Proteins selected for biochemical characterization in this work are shown in red font.

Based on sequence analysis, the 80 validated esterases belong to 17 protein families (Fig. 3). A majority of these enzymes are predicted to belong to the α/β hydrolase superfamily (59 proteins), which represents one of the largest groups of structurally related proteins (148 families in the ESTHER database) with diverse catalytic and non-catalytic functions including hydrolases, dehalogenases, haloperoxidases, and hydroxynitrile lyases^36^,^38^,^42^,^43^. Their catalytic activity depends on the conserved catalytic triad, which consists of a nucleophile (serine, aspartate or cysteine), a histidine and a catalytic acid (aspartate or glutamate). Most α/β hydrolases that we have identified have a conserved Gly-x-Ser-x-Gly catalytic motif and are distributed among eleven different families, with a majority belonging to α/β hydrolase-3, α/β hydrolase-1, and α/β hydrolase-6. The remaining enzymes are distributed among Hydrolase_4, Esterase, Peptidase_S9, COesterase, Chlorophyllase_2, Esterase_phd and DUF676 families, with the exception of two (Fig. 3, Supplementary Fig. S1). Although enzymes MGS0032 and MGS0156 are predicted to belong to the α/β superfamily, they are not associated with known hydrolase families, suggesting that these proteins may belong to new branches.

Two metagenomic esterases, MGS0012 and GEN0034, belong to the DUF3089 family, which appears to be related to α/β hydrolases (Fig. 3, Supplementary Fig. 1). Recently, several members of this family were also isolated from metagenomic libraries and have been shown to exhibit esterase activity^44^,^45^,^46^,^47^,^48^,^49^. Interestingly, MGS0012 shares 99% protein sequence identity with the hypothetical protein WP_026168275 from *Kordiimonas gwangyangensis* (97.6% at the nucleotide level), which has been shown to have the ability to degrade high-molecular weight polycyclic aromatic hydrocarbons^50^.

Ten isolated enzymes belong to the esterase family VIII, which includes β-lactamase-like enzymes with promiscuous β-lactam hydrolytic activity responsible for resistance to β-lactam antibiotics. Previously, several metagenomic β-lactamase-like esterases have been characterized revealing high esterase activity against shorter chain *p*-nitrophenyl esters (C2-C5) and detectable hydrolytic activity against the β-lactamase substrates nitrocefin and cephalosporin^31^,^51^. All ten identified β-lactamase-like esterases are serine hydrolases with a conserved Ser-x-x-Lys catalytic motif typical for the class C β-lactamases. Interestingly, the α/β-hydrolase-like esterase GEN0169 has an additional Metallo-β-lactamase domain (PF00753). This is a domain commonly found in class B β-lactamases, a structurally unrelated enzyme family also capable of hydrolyzing β-lactam antibiotics.

The remaining hydrolase-like proteins share sequence similarity with Patatin-like phospholipases (5 proteins), SGNH-hydrolases (3 proteins), and 3-hydroxybutyrate oligomer hydrolase (one protein, PF10605). The unknown protein MGS0084 has only eight homologous sequences in the Uniprot and non-redundant GenBank databases. A protein sequence alignment of MGS0084 with its homologues shows a conserved Gly-His-Ser-His-(Ala/Gly)-Gly motif, which resembles Gly-x-Ser-x-Gly commonly found in α/β hydrolases suggesting that these proteins may represent a new branch of this superfamily (Supplementary Fig. S1).

MGS0169 belongs to the PF04199 family of putative cyclase-like enzymes, which contain a conserved His-x-Gly-Thr-His-x-Asp-x-Pro-x-His motif predicted to form part of the active site. This motif is only partially conserved in MGS0169 and its closest homologues with the two first His residues replaced by Gln (Supplementary Fig. S1). Several cyclase-like proteins from different bacteria have been shown to exhibit metal-dependent amidohydrolase activity against formylkynurenine and isatin^52^,^53^,^54^, but carboxylesterase activity of PF04199 proteins has not been reported before. Thus, enzymatic screens of metagenomic libraries have revealed carboxylesterases from diverse protein families, including several candidates, which could not have been annotated based on sequence analysis.

### Biochemical characterization of selected metagenomic esterases

**Figure 4:**
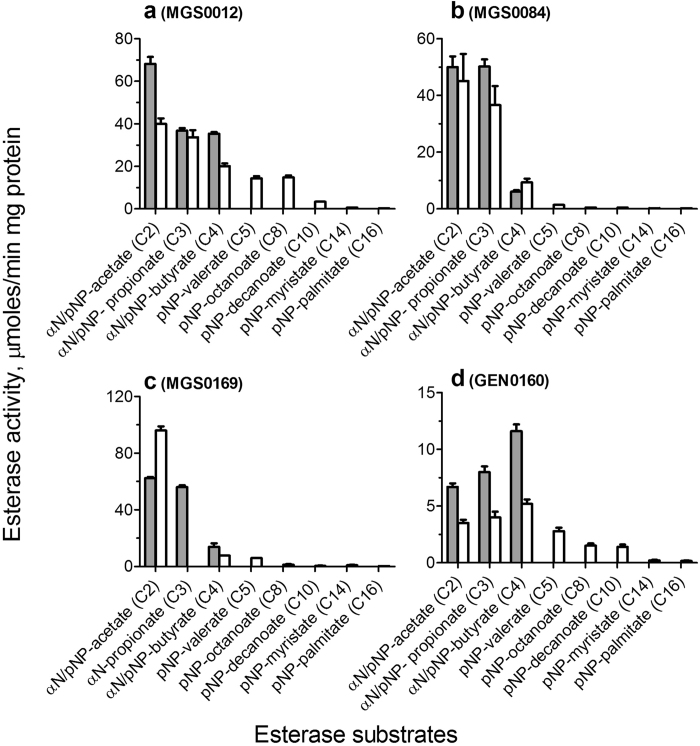
Substrate acyl chain length preference of metagenomic esterases. Esterase activity of purified proteins against *p*-nitrophenyl esters (*p*NP-, white bars) or α-naphthyl esters (αN-, gray bars) with different acyl chain lengths. The reaction mixtures contained the indicated substrate (1 mM) and purified proteins: (**a**) 0.2 μg of MGS0012; (**b**) 0.2 μg of MGS0084; (**c**) 0.025 μg of MGS0169 (and 0.1 mM MnCl_2_); (**d**) 0.3 μg of GEN0160.

For biochemical and structural characterization of metagenomic esterases, we selected the lipolytic enzymes MGS0084 and GEN0160, as well as the novel esterases MGS0012 (DUF3089) and MGS0169 (a cyclase-like protein). The selected proteins were over-expressed in *E. coli* and affinity-purified to over 95% homogeneity. The acyl chain length preference of metagenomic esterases was analyzed using 11 model esterase substrates including three α-naphthyl and eight *p*-nitrophenyl (*p*NP) esters with different chain lengths (Fig. 4). MGS0012 showed the highest activity with α-naphthyl acetate, MGS0169 against *p*NP-acetate, whereas MGS0084 exhibited comparable activity against α-naphthyl- and *p*NP-acetate and propionate (Fig. 4). In contrast, GEN0160 showed a preference to substrates with longer acyl chains with the highest activity against *p*NP-octanoate α-naphthyl- or *p*NP-butyrate (C4, GEN0160). This protein showed detectable esterase activity against *p*NP-palmitate (C16), which is a representative substrate for lipases (Fig. 4). This is in line with the presence of hydrolytic activity of this enzyme toward olive oil (Fig. 1).

In contrast to the other three proteins, esterase activity of MGS0169 was greatly stimulated by the addition of divalent metal cations (Mn^2+^ > Mg^2+^ > Co^2+^ ≫ Ni^2+^) (Supplementary Fig. S2). Several biochemically characterized members of the cyclase-like protein family (PF04199) exhibited metal ion dependent amidohydrolase activity against formylkynurenine and isatin^52^,^53^,^54^. MGS0169 also showed detectable metal dependent amidohydrolase activity against isatin (*k*_cat_/*K*_M_ 0.1 × 10^3^ M^−1^ s^−1^), but its esterase activity against *p*NP-acetate was at least three orders of magnitude higher (*k*_cat_/*K*_M_ ~ 0.2 × 10^6^ M^−1^ s^−1^) (Fig. 4, Table 2). Previously, the presence of metal ion-stimulated esterase activity was demonstrated in the amidohydrolase proteins from the phosphotriesterase family (PF02126) including Rsp3690 from *Rhodobacter sphaeroides* and Pmi1525 from *Proteus mirabilis*^55^,^56^. However, these enzymes have different structural folds and active sites. Thus, MGS0169 and homologous proteins from the PF04199 family do indeed represent a novel group of metal-dependent esterases from the amidohydrolase superfamily.

The purified metagenomic esterases showed saturation kinetics and high catalytic efficiencies with low *K*_M_ values toward the tested model esterase substrates (Table 2). The ester substrate profiles of four metagenomic esterases were determined using a library of 89 various monoesters including alkyl and aryl esters (Supplementary Fig. S3, Supplementary Table S3). These proteins showed hydrolytic activity against a broad range of substrates with different substrate preferences. MGS0012, MGS0169 and GEN0160 were most active against phenyl acetate, whereas MGS0084 against vinyl laurate (Supplementary Fig. S3). From these proteins, MGS0012 was found to be the most efficient esterase showing high *k*_cat_/*K*_M_ values toward a broad range of substrates including the medium acyl chain esters (C4-C10) (Table 2).

The esterase activities of metagenomic esterases showed different temperature profiles determined in the range from 5 °C to 70 °C (Supplementary Fig. S4). MGS0084 was most active at 25 °C but retained almost 50% of maximal activity at 5 °C suggesting that it is a cold-adapted enzyme. In contrast, the other metagenomic esterases showed maximal activity at 40 °C and retained less than 15% of maximal activity at 5 °C, which is typical of mesophilic enzymes (Supplementary Fig. S4). The esterases also showed different sensitivities to high salt concentrations with MGS0084 exhibiting strong inhibition by 0.25 M NaCl or KCl (Supplementary Fig. S5). In contrast, the esterase activity of MGS0012 and GEN0160 was slightly stimulated by the addition of salt, and they showed no inhibition even at 2 M or 3 M salt concentration (Supplementary Fig. S5). The metagenomic esterases also showed different sensitivities to solvents (acetonitrile and DMSO) with MGS0012 being the most sensitive enzyme and GEN0160 being the most resistant enzyme (Supplementary Fig. S5). Thus, the characterized metagenomic esterases exhibit different temperature profiles and sensitivities to inhibition by salt and solvents perhaps reflecting differences in native environmental conditions and hosts.

### Polyester depolymerization activity of purified metagenomic esterases

**Figure 5:**
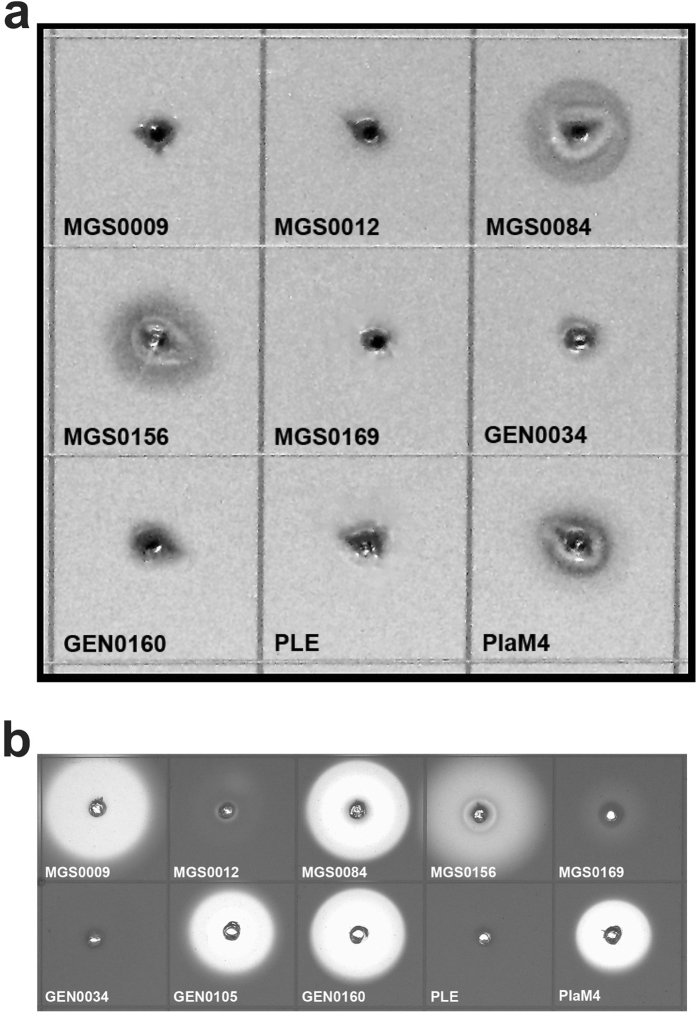
Hydrolytic activity of purified metagenomic esterases against polyester substrates. Agarose-based polyester depolymerase screens with emulsified PLA2 (**a**) or polycaprolactone PCL10 (**b**). Agarose gels (1.5%) contained 0.2% PLA2 or PCL10 emulsified in 50 mM Tris-HCl (pH 8.0) containing 0.01% Plysurf A210G. The wells in agarose gel were loaded with 50 μg of purified proteins including the positive (PlaM4) and negative (PLE) controls. The formation of a clear halo around the wells is attributed to the enzymatic hydrolysis of insoluble polymeric substrates.

Recent studies including our work have demonstrated the presence of hydrolytic activity against polylactic acid (PLA), a biodegradable polyester, in several lipolytic enzymes and carboxylesterases^31^,^57^,^58^. In this work, 26 purified metagenomic esterases were screened for PLA-degrading activity using an agarose plate assay with the emulsified PLA2 (M_w_ 2 K). These screens revealed the presence of PLA hydrolytic activity in seven enzymes including the lipolytic esterases MGS0084 and MGS0156 (Fig. 5). An agarose-based screening of purified esterases using the emulsified polycaprolactone PCL10 (M_w_ 10 K), another biodegradable polyester, demonstrated the presence of high PCL10 depolymerization activity in MGS0084, GEN0105, and GEN0160, as well as in MGS0009 and MGS0156 (Fig. 5). The hydrolytic activity of the identified metagenomic esterases against different polyester substrates makes these enzymes attractive candidates for studies toward enzyme-based depolymerization of polyester plastics.

### Crystal structure of the metal ion dependent esterase MGS0169

**Figure 6:**
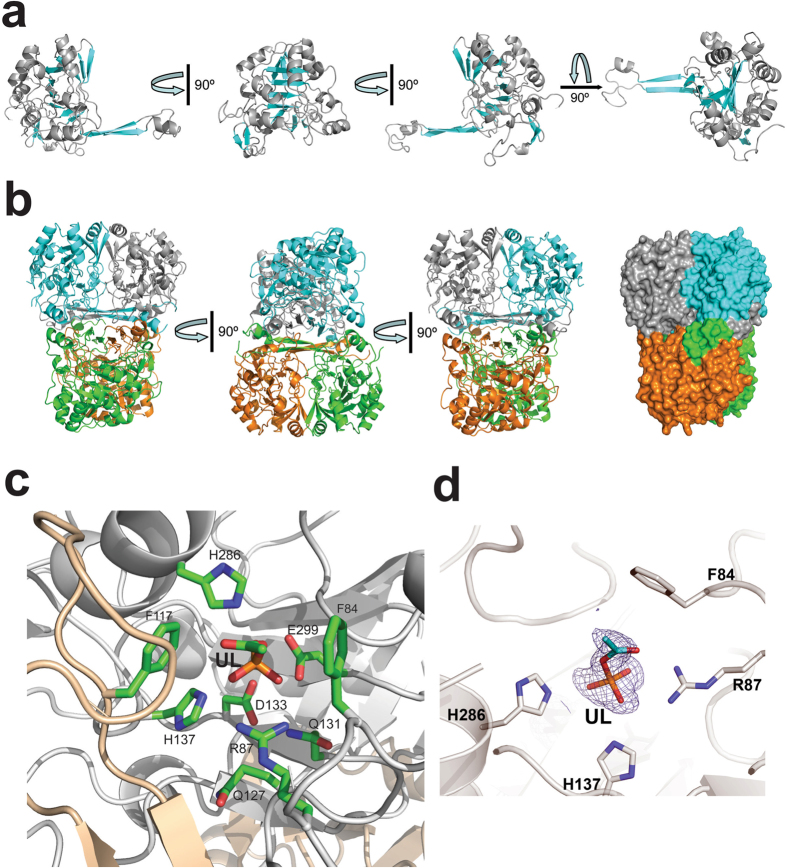
Crystal structure of MGS0169. (**a**) Overall structure of the MGS0169 protomer (several orientations related by 90° rotation). The ribbon diagram of the core domain is colored gray (helices) and cyan (β-strands). (**b**) Three views of the MGS0169 tetramer related by 90° rotation with monomers colored gray, cyan, green, and orange. The last view is also shown in a surface presentation to demonstrate the tight packing of monomers. (**c**) Close-up view of the MGS0159 active site showing the bound unknown ligand (UL). The amino acid side chains and ligand molecule are shown as sticks along a protein ribbon colored gray. In the MGS0169 structure, the second protomer (colored wheat) contributes to the substrate binding site of the first protomer (shown in gray).

The purified seleno-methionine-substituted metagenomic esterases were also submitted to crystallization trials. MGS0169 (21–341 aa) produced diffracting crystals, and its crystal structure was determined at 1.61 Å resolution (Supplementary Table 4). The MGS0169 protomer core has a slightly distorted central β-barrel containing both parallel and anti-parallel β-strands surrounded by ten α-helices, whose fold resembles the swivelling β/α/β domain of metal-dependent α/β hydrolases^53^,^59^. The small sub-domain of MGS0169 is comprised two β-strands (β2 and β3) connected by a flexible loop containing a short α-helix with the strands of one protomer forming a four-stranded anti-parallel β-sheet with the two related β-strands of another protomer (Fig. 6). This results in the formation of a tightly packed twisted (~90°) tetramer through the interaction between the β-sheets stabilized by interactions between the surrounding α-helices (Fig. 6). Analysis of the crystal contacts using the quaternary structure prediction server PISA suggests that MGS0159 is likely to form tetramers through multiple interactions between tightly packed monomers burying ~7,000 Å^2^ of the solvent accessible surface per monomer (~30% of the total solvent accessible surface). The tetrameric organization of MGS0169 is supported by the results of size-exclusion chromatography suggesting a trimeric or tightly packed tetrameric organization (112 kDa, predicted Mw 37 kDa).

A Dali search for MGS0169 structural homologues identified several protein structures as the best matches including the isatin hydrolase IH-b from *Labrenzia aggregata* (PDB codes 4J0N and 4M8D; Z-score 21.3, rmsd 2.5 Å), the three microbial kynurenine formamidases KynB (PDB codes 4COB, 4COG, and 4CO9; Z-score 19.4–19.9, rmsd 2.1–2.7 Å), and the uncharacterized predicted hydrolase P84132_GEOSE from *Geobacillus stearothermophilus* (PDB code 3KRV; Z-score 18.6, rmsd 2.9 Å). These proteins share low sequence similarity to MGS0169 (17–22% sequence identity), and the two biochemically characterized enzymes (IH-b and KynB) have metal-dependent amidohydrolase activity against isatin and N-formylkynurenine^53^,^54^.

### The active site of MGS0169

The location of the MGS0169 active site is indicated by the unknown electron density with a tetrahedral-like geometry located in the narrow cavity formed mainly by α-helices near the end of the central β-barrel (Fig. 6, Supplementary Fig. S2). Based on its shape, this density might represent a molecule of acetyl-phosphate, probably captured by the enzyme from *E. coli* cells. The bottom part of the ligand is positioned close to the side chains of the conserved Gln127 (5.3 Å), Gln131 (4.3 Å), Asp133 (3.8 Å), and His137 (2.6 Å) (Fig. 6). These residues represent a modified cyclase-like motif Gln-x-x-x-Gln-x-Asp-x-x-x-His found in several cyclase-like proteins (PF04199) including the *trans*-dienelactone hydrolase from *Pseudomonas reinekei* MT1^60^ and are likely to be involved in metal ion binding. The bound ligand also interacts with the side chains of the conserved Arg87 (2.9 Å), Glu299 (2.6 Å), and His286 (2.6 Å). The MGS0169 substrate binding site is less conserved with only two residues (Phe84 and His286) identical to the isatin hydrolase substrate binding site (Phe41 and His212, PDB code 4J0N) (Fig. 6).

In the crystal structures of isatin hydrolase IH-b (PDB code 4M8D) and kynurenine formamidase KynB (PDB code 4COB), which contain the classical cyclase-like motif His-x-x-x-His-x-Asp-x-x-x-His, the side chains of the motif residues coordinate one Mn^2+^ ion (IH-b) or two Zn^2+^ ions (KynB), which are required for the enzymatic activity of these enzymes^53^,^54^. However, no metal ion was found in the corresponding site of the uncharacterized cyclase-like protein MJ0783 from *Methanocaldococcus jannaschii* (PDB code 2B0A), or in MGS0169. We propose that metal ion binding to MGS0169 was prevented by the presence of the bound acetylphosphate-like ligand, and the active site of the catalytically active MGS0169 contains one or two metal ions (probably Mn^2+^, based on the MGS0169 metal ion profile), as was suggested for the the *trans*-dienelactone hydrolase from *Pseudomonas reinekei* MT1^61^.

The structure of the MGS0169 dimer also revealed that the side chain of Phe117 (and possibly Phe110 and Lys112) from one protomer contributes to the substrate binding site of another protomer and is positioned near the bound ligand (3.7 Å) and side chains of His137 (3.6 Å) and His286 (4.0 Å) (Fig. 6). This suggests that the two active sites of the MGS0169 dimer can allosterically interact through the residues of the composite binding sites, which is in line with the observed range of Hill coefficients of 1.2–1.5. The other highly conserved residues of the MGS0169 active site, which can potentially contribute to substrate binding include Phe84 (4.6 Å to the acetyl-like ligand), Arg87 (2.9 Å to the ligand), His286 (2.5 Å), and Glu299 (2.6 Å). Thus, the active site residues of MGS0169 and other cyclase-like proteins are different from those of non-specific carboxylesterases from the amidohydrolase superfamiliy^55^,^56^.

### Validation of the catalytic residues of metagenomic esterases using site-directed mutagenesis

**Figure 7:**
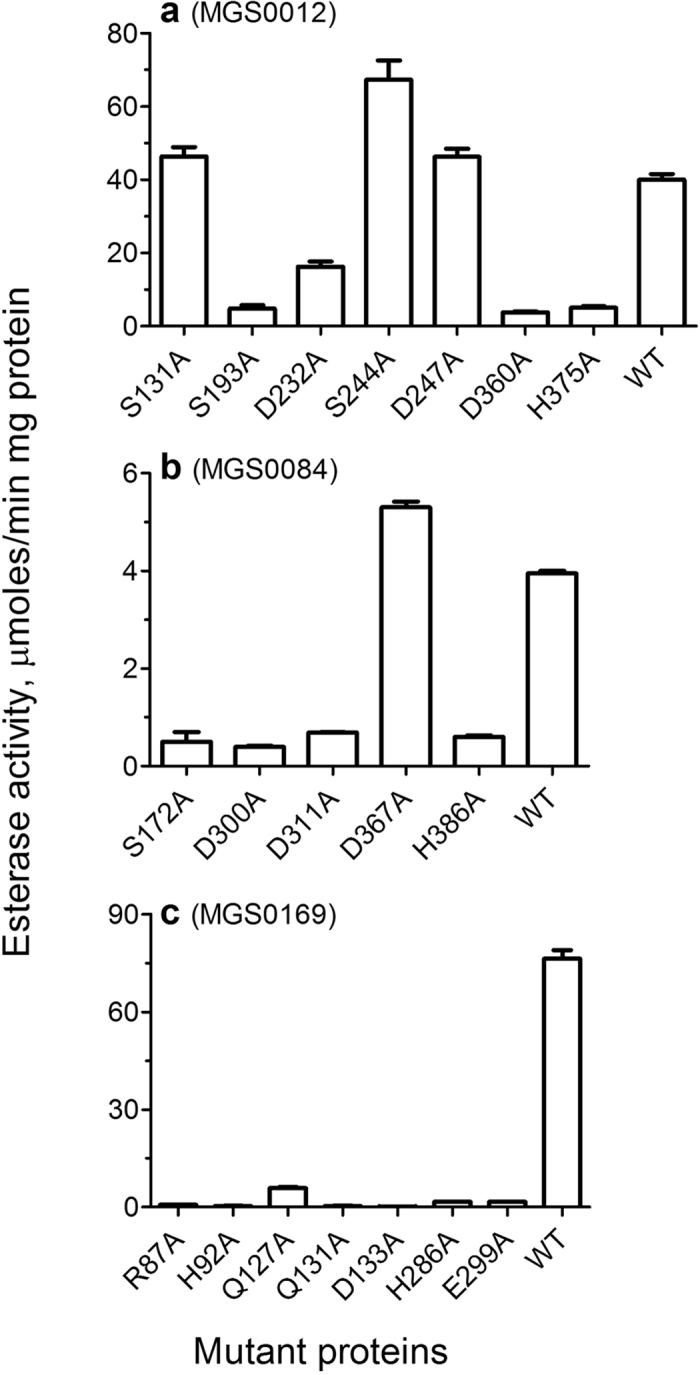
Identification of catalytic residues of novel esterases using site-directed mutagenesis: esterase activity of purified mutant proteins. The reaction mixtures contained: (**a**) 2 mM α-naphthyl acetate and 0.2 μg of MGS0012; (**b**) 1.5 mM α-naphthyl propionate and 2 μg of MGS0084; (**c**) 1.5 mM *p*NP-acetate, 0.1 mM MnCl_2_, and 0.03 μg of MGS0169.

The potential active site residues of metagenomic esterases, selected based on their sequence alignments (Supplementary Fig. S1) and the MGS0169 crystal structure (Fig. 6), were verified using site-directed mutagenesis (alanine replacement). The mutant proteins were purified using affinity chromatography, and their enzymatic activity was compared to that of wild type proteins. As shown in Fig. 7, the catalytic triad of the DUF3089 hydrolase MGS0012 includes Ser193, Asp360, and His375, because the corresponding mutant proteins showed a greatly reduced catalytic activity. Low activity was also found in the MGS0012 D232A mutant protein, whereas S131A, S244A, and D247A retained high enzymatic activity (Fig. 7). Similarly, alanine replacement mutagenesis of the unknown protein MGS0084 suggested that it is a novel Ser-dependent hydrolase with the catalytic triad comprising Ser172, Asp300, and His386 (Fig. 7).

Alanine replacement mutagenesis of the metal-dependent esterase MGS0169 (from the cyclase-like family) revealed that this enzyme is sensitive to mutations in the active site including the residues of the modified cyclase-like motif Gln127, Gln131, and Asp133 (H137A was insoluble), as well as those involved in substrate binding (Arg87, H286, and Glu299) (Fig. 7). Thus, site-directed mutagenesis of metagenomic esterases revealed that MGS0012 and MGS0084 represent novel Ser-dependent hydrolases, whereas MGS0169 is a novel metal-dependent esterase with the modified cyclase-like motif Gln127-Gln131-Asp133-His137 potentially involved in metal ion binding.

## Conclusions

The discovery of new enzymes in environmental bacteria contributes greatly to our fundamental knowledge of protein structure-function relationships, expands the biocatalytic toolbox of enzymes for metabolic engineering and synthetic biology, and improves the quality of gene annotation in public databases, on which bioinformatics tools rely. Enzymatic screening of environmental gene libraries presented in this work revealed a huge sequence and biochemical diversity with identification of 80 esterases from 17 different enzyme families (Fig. 3). These enzymes exhibit diverse substrate preferences, from short to long acyl chain esters with a significant number of enzymes possessing polyesterase activity against polylactic acid and polycaprolactone. The differences in their sensitivities to temperature and salt conditions likely reflect environmental adaptations. Through activity-based screening, we have been able to identify three novel Ser-dependent esterases and present the crystal structure of a new carboxylesterase subfamily within the cyclase-like family of metal ion dependent amidohydrolases. This work contributes a 30% increase in experimentally validated metagenomic esterases (288 enzymes according to a recent analysis^35^). By combining metagenomic enzyme discovery with protein and metabolic engineering, we may gain access to virtually unlimited diversity of enzyme sequences, with the potential to discover tailor-made enzymes for any biotransformation reaction.

## Methods

### Metagenome library preparation, gene cloning, and protein purification

Extraction of metagenomic DNA from environmental samples and preparation of fosmid and lambda-ZAP DNA libraries (Supplementary Table S1) were performed as described previously^25^,^62^,^63^,^64^. Genes were amplified by PCR from purified fosmids or excised lambda-ZAP plasmids and cloned into a modified pET15b vector, containing an N-terminal 6His tag, as described previously^65^. Tagged genes were overexpressed in *E. coli* BL21(DE3) and affinity purified using metal-chelate chromatography on Ni-NTA (Qiagen). Site-directed mutagenesis of selected enzymes was performed based on the QuikChange^®^ site-directed mutagenesis kit (Stratagene). All mutations were verified by DNA sequencing, and the mutant proteins were overexpressed and purified like the wild-type.

### Enzymatic screening of metagenomic libraries

*E. coli* fosmid clones were cultured at 37 °C in 384-well microtiter plates, and spotted onto Luria Broth (LB) agar plates containing chloramphenicol (12.5 μg/mL), arabinose (0.001–0.01%), gum Arabic (0.5%), and emulsified tributyrin (1%). Clones were grown overnight at 37 °C, then at 30 °C for 3–4 days. Colonies with clear halos were considered positive for esterase activity, and selected for plasmid extraction. Lambda-ZAP clones were screened as follows. 300 μL of mid-log phase *E. coli* XL1-Blue MRF’ cells were infected the Lambda-ZAP library added to 4 mL of 0.7% LB agar containing 10 mM MgSO_4_, 1 mM IPTG, 0.5% gum arabic and 1% of emulsified tributyrin, at 48 °C. The mixture was immediately layered onto LB agar plates containing 1 mM IPTG, at approximately 1,000 plaque forming units per plate, and the plates were incubated at 37 °C. Phage plaques exhibiting a clear halo over 3–4 days were isolated, and plasmids containing the metagenomic segments were extracted from phage DNA according to the manufacturer’s protocol.

To confirm esterase activity in cloned proteins, *E. coli* expressing the cloned genes were streaked onto LB-agar plates containing 1% tributyrin (as above) or purified enzymes were spotted directly and the plates were incubated at 30 °C or 37 °C. Clones were also checked for lipase activity either by streaking *E. coli* colonies or spotting 5–10 μg of the purified enzymes onto LB agar plates containing 3% emulsified olive oil and 0.001% Rhodamine B indicator dye, and incubating at 30 °C or 37 °C. Lipase activity was identified under UV light as orange fluorescence^66^.

### Sequencing of metagenomic fragments and bioinformatics analysis

Lambda-ZAP clones were sequenced by primer walking, while fosmids were sequenced as mixed pools using Illumina or Roche 454 platforms (at TCAG, Genome Quebec and Genoscope). Reads were dereplicated and assembled into contigs using the Velvet algorithm^67^ in Geneious^68^ version 6.0.6, and contigs were mapped to specific fosmids using Sanger sequenced fosmid end sequences. Contigs were submitted to the MG-RAST^69^ pipeline for gene annotation. In parallel, open reading frames were predicted using the Glimmer algorithm^70^, and translated protein sequences were annotated through BLAST searches of UniProt and the non-redundant GenBank protein database^71^. Genes predicted as esterases, lipases, or hydrolases were selected for recombinant expression in *E. coli.* Where no such gene was found, smaller esterase positive genetic fragments were identified by subcloning, and all predicted genes were cloned and rescreened.

Proteins with confirmed esterase activity (Supplementary Table S2) were classified into families through sequence analysis using HMMER^72^ searches against the Pfam database and BLAST searches of the COG database^73^, with an E-value cut-off of 1E-5 unless otherwise indicated. Where an enzyme had a significant score to more than one protein family, the family with the smaller E-value and/or larger sequence coverage was assigned. Multiple sequence alignments were generated using MUSCLE^74^. The phylogenetic tree was produced using the NCBI taxonomy of the closest sequence homologues and the PhyloT tree generator (http://phylot.biobyte.de/) and visualized using the iTOL v3 online tool^75^.

### Enzymatic assays with purified proteins

Carboxylesterase activity of purified proteins against *p*-nitrophenyl (*p*NP) or α-naphthyl esters of various fatty acids was measured spectrophotometrically as described previously^31^. The effect of temperature, salts, and solvents on esterase activity of purified proteins against the indicated α-naphthyl substrate was measured using the same protocol. Hydrolytic activity of purified enzymes against a library of 89 ester substrates was determined spectrophotometrically using *p*-nitrophenol as described previously^31^. Depolymerization activity of purified enzymes against polylactic acid (PLA) or polycaprolactone (PCL) was determined essentially as described previously^31^. These assays were performed in agarose plates (1.5%) containing emulsified substrates (poly (DL-lactide), average M.W. 2,000, or PCL10), or in solution (20 mg of PLA10 in 1 ml of 0.4 M Tris-HCl buffer, pH 8.0, 0.01% Plysurf A210G) at 32 °C. For determination of kinetic parameters (*K*_M_ and *k*_cat_), esterase activity was determined over a range of substrate concentrations (0.01–5.0 mM). Kinetic parameters were calculated by non-linear regression analysis of raw data fit (to the Michaelis-Menten or Hill functions) using GraphPad Prism software (version 4.00 for Windows).

### Crystallization and structure determination of MGS0169

The selenomethionine substituted MGS0169 (21–341 aa) was crystallized at 22 °C using the sitting-drop vapor diffusion method by mixing 0.5 μl of the purified protein (20 mg/ml) with 0.5 μl of the crystallization solution containing 0.2 M ammonium acetate, 0.1 M Tris-HCl (pH 8.0), and 30% (w/v) PEG 2KMME. The crystals were stabilized by cryoprotection in Paratone-N prior to flash-freezing in liquid nitrogen. Diffraction data were collected at the beamline 19-ID with an ADSC Quantum 315 R detector of the Structural Biology Center, Advanced Photon Source, Argonne National Laboratory^76^,^77^. Diffraction data were processed using the HKL3000 suit of programs^78^, and structural statistics is summarized in Supplementary Table S4. The MGS0169 structure was determined by the single-wavelength anomalous diffraction (SAD) method using phasing, density modification, and initial protein model building as implemented in the HKL3000 software package^79^,^80^,^81^,^82^,^83^,^84^. Several cycles of manual corrections of the model were carried out using the programs COOT^85^ and REFMAC of the CCP4^86^ and finalized using Phenix^87^. The final model was refined against all reflections except for 5% randomly selected reflections, which were used for monitoring *R*_free_. The final refinement statistics are presented in Supplementary Table S4.

## Additional Information

**Accession codes:** The sequences of 80 metagenomic esterases identified and verified in this project have been submitted to GenBank with the accession numbers shown in Supplementary Table S2. The atomic coordinates and structure factors for the MGS0169 structure have been deposited in the RCSB Protein Data Bank with accession code 5IBZ.

**How to cite this article**: Popovic, A. *et al*. Activity screening of environmental metagenomic libraries reveals novel carboxylesterase families. *Sci. Rep.* **7**, 44103; doi: 10.1038/srep44103 (2017).

**Publisher's note:** Springer Nature remains neutral with regard to jurisdictional claims in published maps and institutional affiliations.

## Supplementary Material

Supplementary Information

## Figures and Tables

**Table 1:** Metagenomic libraries used in this work and esterase screening results.

Metagenome library	Number of clones in library	Clones screened	Positive clones		Validated esterases^a^
			Identified	Sequenced	
1. Anaerobic waste water digester (Evry, France)	99,840	47,616^c^	254	110	28
2. Composting plant (Liemehna, Germany)	49,000	60,000^b^	6	6	5
3. Kolguev Island (Barents Sea)	100,000	142,000^b^	34	34	4
4. Messina harbour (Mediterranean Sea)	1,000	24,000^b^	18	18	8
5. Messina Int II (Mediterranean Sea)	10,368	5,760^c^	208	50	1
6. Michle soil (Czech Republic)	15,000	99,900^b^	20	20	3
7. Milazzo (Mediterranean Sea)	2,400	20,000^b^	8	8	3
8. MT Haven sunken shipwreck (Ligurian Sea, Italy)	25,000	36,800^b^	19	19	11
9. Priolo (Gargallo, Italy)	40,000	118,500^b^	4	4	2
10. Port of Murmansk (Barents Sea)	100,000	108,000^b^	43	43	3
11. *Rimicaris exoculata* gill	150,000	21,100^b^	5	5	2
12. *Rimicaris exoculata* gut	350,000	137,500^b^	4	4	2
13. Sobeslav soil (Czech Republic)	2,500	114,000^b^	5	5	2
14. Tembec Paper Mill (Ontario, Canada)	100,000	53,500^b^	1	1	1
15. Urania DHAL (Mediterranean Sea)	100,000	90,800^b^	41	41	1
16. Vulcano Island (Mediterranean Sea)	3,456	1,920^c^	44	18	1
17. *Cycloclasticus* sp. 78-ME (genomic library)	768	768^c^	27	24	3
Total		**1,080,628**	**714**	**386**	**80**

^a^Esterase activity of selected proteins was confirmed by subcloning and individual expression in *E. coli* followed by assays with crude extracts or purified proteins.

^b^Lambda-ZAP libraries, mixed clone pools.

^c^Fosmid libraries.

**Table 2:** Kinetic parameters of purified metagenomic esterases.

Protein	Variable substrate	*K*_M_, *mM*	*k*_cat_, *s*^*−1*^	*k*_cat_/*K*_M_, *M*^*−1*^*s*^*−1*^
MGS0012	α-NA^a^ (C2)	0.71 ± 0.04	51.0 ± 1.0	0.7 × 10^5^
	β-NA (C2)	0.41 ± 0.05	25.3 ± 1.0	0.6 × 10^5^
	α-NP (C3)	0.11 ± 0.01	28.8 ± 0.4	2.6 × 10^5^
	α-NB (C4)	0.10 ± 0.01	29.5 ± 0.5	3.0 × 10^5^
	*p*NP-acetate (C2)	2.12 ± 0.29	33.7 ± 2.3	0.2 × 10^5^
	*p*NP-propionate (C3)	1.62 ± 0.31	28.1 ± 2.8	0.2 × 10^5^
	*p*NP-butyrate (C4)	1.46 ± 0.19	16.8 ± 1.2	0.1 × 10^5^
	*p*NP-valerate (C5)	0.87 ± 0.12	12.1 ± 0.8	0.1 × 10^5^
	*p*NP-octanoate (C8)	0.78 ± 0.09	12.3 ± 0.8	0.2 × 10^5^
	*p*NP-decanoate (C10)	0.23 ± 0.02	2.8 ± 0.1	0.1 × 10^5^
MGS0084	α-NA (C2)	0.71 ± 0.11	39.8 ± 2.9	0.6 × 10^5^
	α-NP (C3)	0.43 ± 0.04	39.8 ± 2.0	0.9 × 10^5^
	*p*NP-acetate (C2)	4.1 ± 1.2	35.8 ± 8.0	0.8 × 10^4^
	*p*NP-butyrate (C4)	2.4 ± 0.5	7.4 ± 1.1	0.3 × 10^4^
	*p*NP-valerate (C5)	0.53 ± 0.06	1.1 ± 0.1	0.2 × 10^4^
GEN0160	α-NA (C2)	1.07 ± 0.04	6.1 ± 0.2	0.6 × 10^4^
	α-NP (C3)	0.65 ± 0.03	7.3 ± 1.7	0.1 × 10^5^
	α-NB (C4)	0.32 ± 0.04	10.6 ± 0.5	0.3 × 10^5^
MGS0169	α-NA (C2)	0.49 ± 0.11	14.9 ± 1.2	0.3 × 10^5^
	α-NP (C3)	0.42 ± 0.12	14.7 ± 1.4	0.4 × 10^5^
	*p*NP-acetate (C2)	0.40 ± 0.29	71.2 ± 1.7	1.8 × 10^5^
	isatin (amidohydrolase activity)	19.7 ± 4.2	1.9 ± 0.2	0.1 × 10^3^

^a^α-NA, α-naphthyl acetate; β-NA, β-naphthyl acetate; α-NB, α-naphthyl butyrate; α-NP, α-naphthyl propionate.
