# Supplementary material for: Activity screening of environmental metagenomic libraries reveals novel carboxylesterase families
Source: Sci Rep. 2017 Mar 8;7:44103. doi: 10.1038/srep44103 (PMC5341072; doi:10.1038/srep44103)
Supplement: Supplementary Information [file srep44103-s1.pdf]

Supplementary Information

"Activity screening of environmental metagenomic libraries reveals novel carboxylesterase families"

Ana Popovic[1], Tran Hai[2], Anatoly Tchigvintsev[1], Mahbod Hajighasemi[1], Boguslaw Nocek[3], Anna N. Khusnutdinova[1], Greg Brown[1], Julia Glinos[1], Robert Flick[1], Tatiana Skarina[1], Tatyana N. Chernikova[2], Veronica Yim[1], Thomas Brüls[4], Denis Le Paslier[5], Michail M. Yakimov[6], Andrzej Joachimiak[3], Manuel Ferrer[7], Olga V. Golyshina[2], Alexei Savchenko[1], Peter N. Golyshin[2], and Alexander F. Yakunin[1]

[1] Department of Chemical Engineering and Applied Chemistry, University of Toronto, Toronto, ON, M5S 3E5, Canada
[2] School of Biological Sciences, Bangor University, Gwynedd LL57 2UW, UK
[3] Midwest Center for Structural Genomics and Structural Biology Center, Biosciences Division, Argonne National Laboratory, Argonne, Illinois 60439, U.S.A.
[4] Commissariat à l'Energie Atomique et aux Energies Alternatives (CEA), Direction de la Recherche Fondamentale, Institut de Génomique, Université de d'Evry Val d'Essonne (UEVE), Centre National de la Recherche Scientifique (CNRS), UMR8030, Génomique métabolique, Evry, France
[5] Université de d'Evry Val d'Essonne (UEVE), Centre National de la Recherche Scientifique (CNRS), UMR8030, Génomique métabolique, Commissariat à l'Energie Atomique et aux Energies Alternatives (CEA), Direction de la Recherche Fondamentale, Institut de Génomique, Evry, France
[6] Institute for Coastal Marine Environment, CNR, 98122 Messina, Italy
[7] Institute of Catalysis, CSIC, Madrid 28049, Spain

Supplementary Figure Legends

Supplementary Figure S1. Protein sequence alignments of metagenomic esterases. (**A**), MGS0012 and GEN0034; (**B**), MGS0084; (**C**), MGS0169; (**D**), GEN0160. Alignments were carried out in Geneious version 6.0.6, using the ClustalW algorithm and BLOSUM scoring matrix. Underlined sequences (orange) indicate conserved motifs and putative catalytic residues.

Supplementary Figure S2. MGS0169: activity metal profile and the active site with unknown ligand. (A), Effect of different divalent metal cations on the esterase activity of MGS0169. The activity was measured in the presence of 1.5 mM *p*NP-acetate, 0.5 mM metal cation (as indicated), and 0.03 µg of MGS0169 (20 min incubation at 30 °C). (**B**), Close-up view of the MGS0169 active site showing the bound unknown ligand (UL) and interacting residues (drawn in stick representation and labeled). Electron density for the ligand is shown using an $F_0 - F_C$ map contoured at 3.0 σ.

Supplementary Figure S3. Activity profiles of purified metagenomic esterases against different ester substrates. The activity was assayed against a library of 89 ester substrates (only substrates with significant esterase activity are shown). The reaction mixtures (100 µl) contained 2 mM BES buffer (pH 7.2), 2 mM substrate, 0.45 mM *p*-nitrophenol, and 0.25-2.0 µg of enzyme (also 0.1 mM $MnCl_2$ for MGS0169).

Supplementary Figure S4. Effect of assay temperature on enzymatic activity of purified metagenomic esterases. Esterase activity was measured at indicated temperatures using 1.5 mM α-naphthyl acetate (A and B), 1.5 mM α-naphthyl butyrate (C and F), 1.5 mM *p*NP-acetate (D), or 1.5 mM α-naphthyl propionate (E) as substrate and purified proteins (0.01 - 0.3 µg).

Supplementary Figure S5. Effect of salts and solvents on enzymatic activity of purified metagenomic esterases. Esterase activity was measured at 30 °C using 1.5 mM α-naphthyl acetate (MGS0012 and MGS0084) or 1.5 mM α-naphthyl butyrate (GEN0160) as substrate and purified proteins (0.01 - 0.3 µg).

Supplementary Figure S1

A

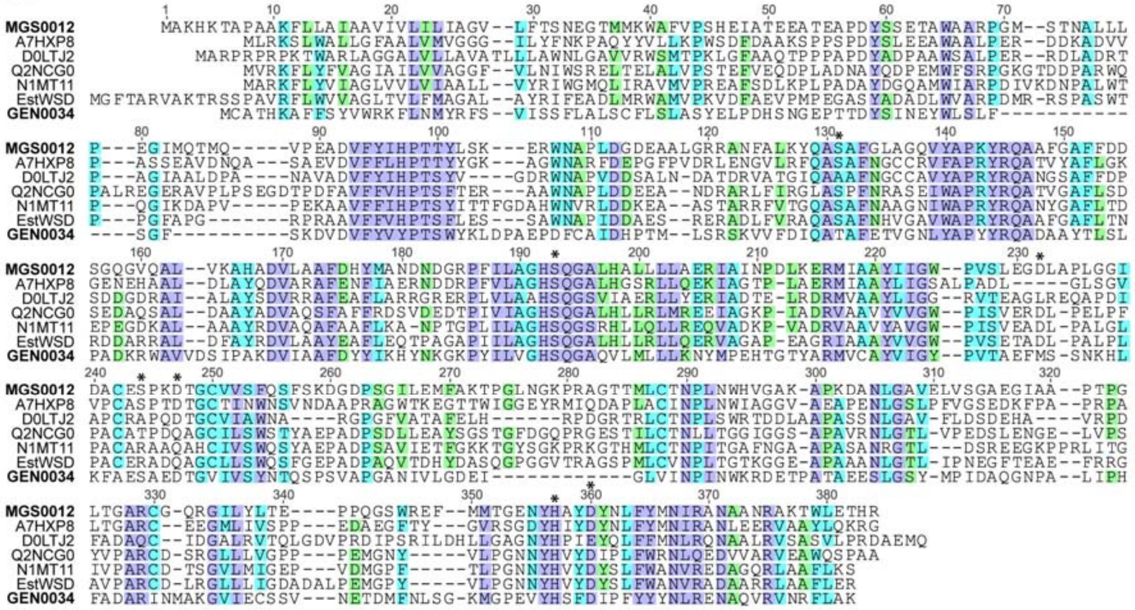

B

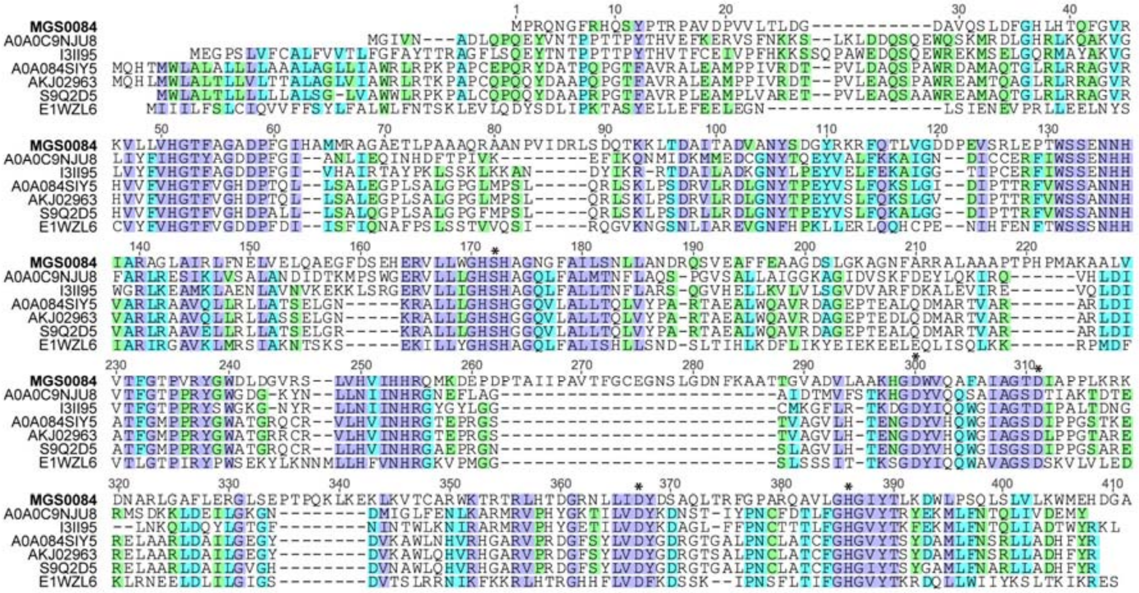

C

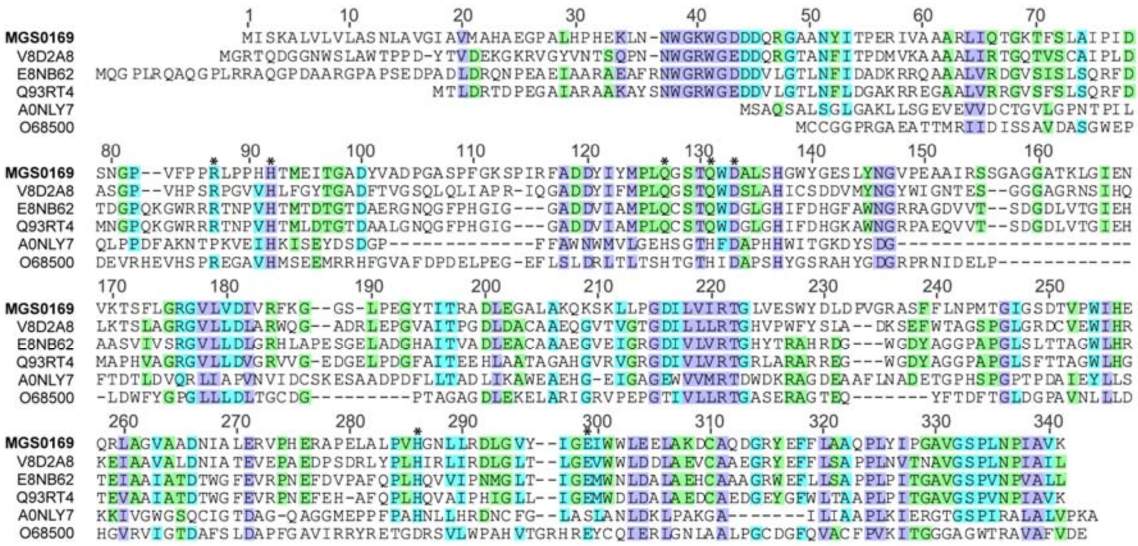
MGS0169
V8D2A8
E8NB62
Q93RT4
A0NLY7
O68500

D

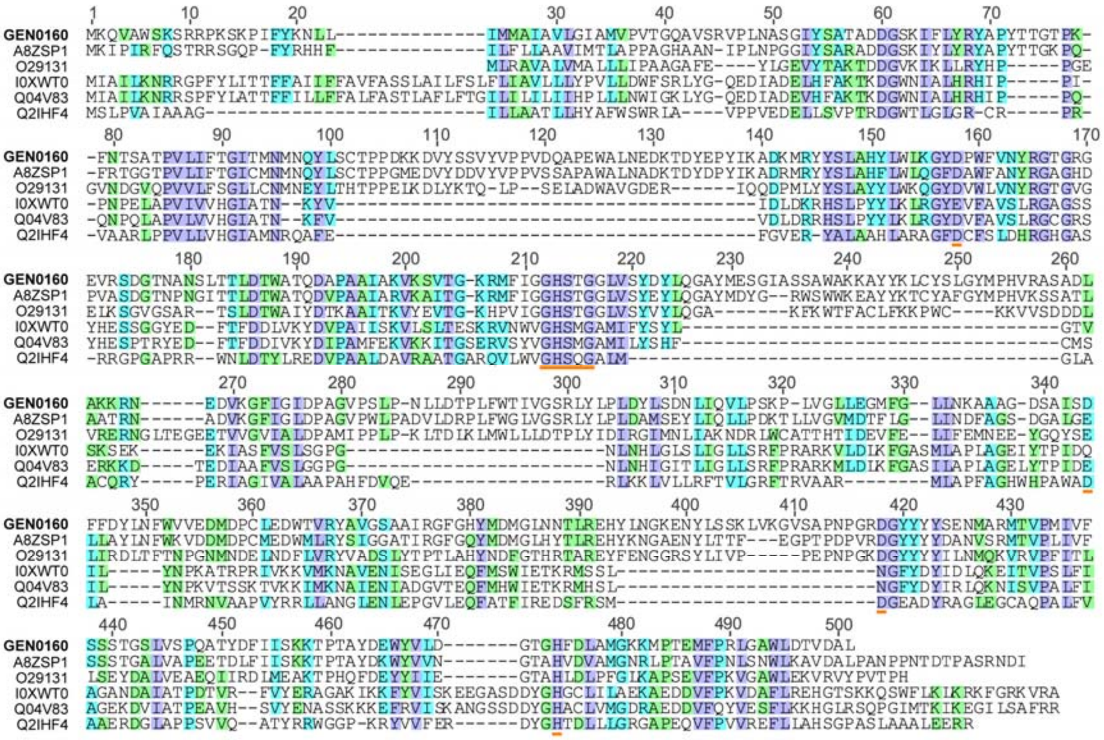
GEN0160
A8ZSP1
O29131
I0XWT0
Q04V83
Q2IHF4

Supplementary Figure S2

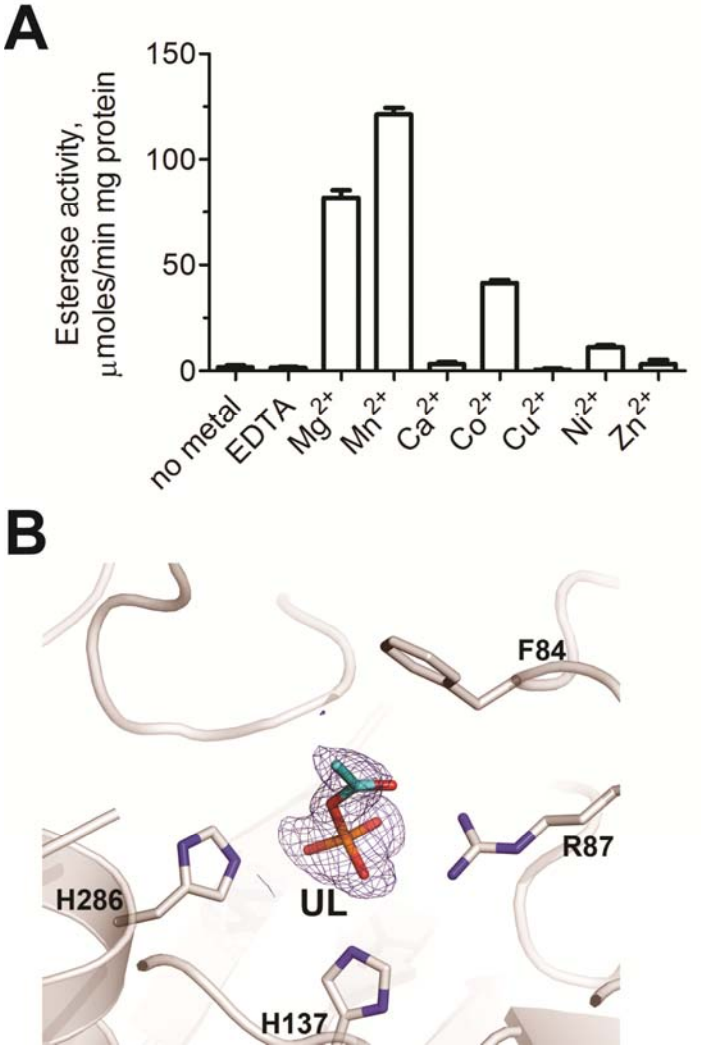

Supplementary Figure S3

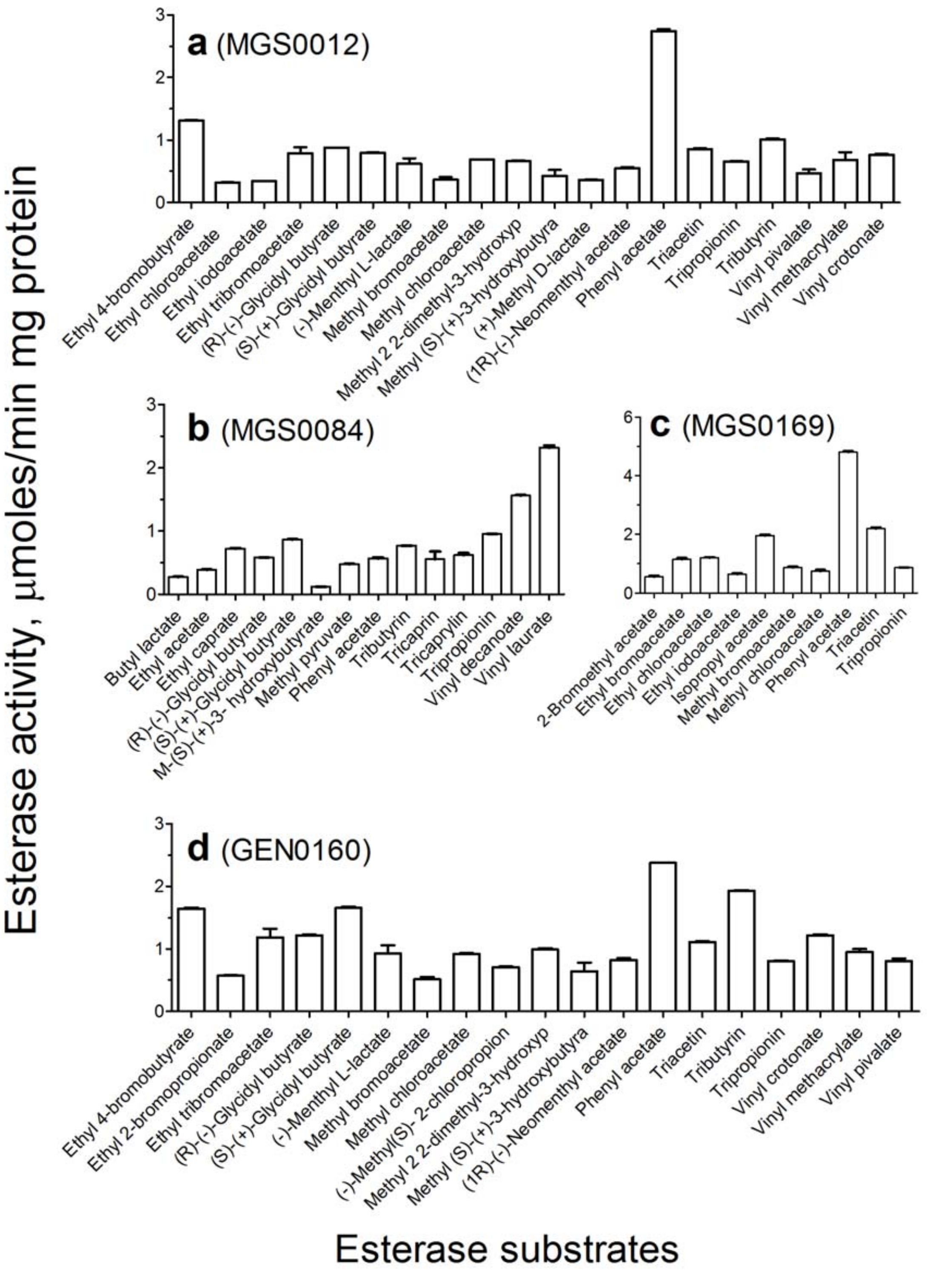

Supplementary Figure S4

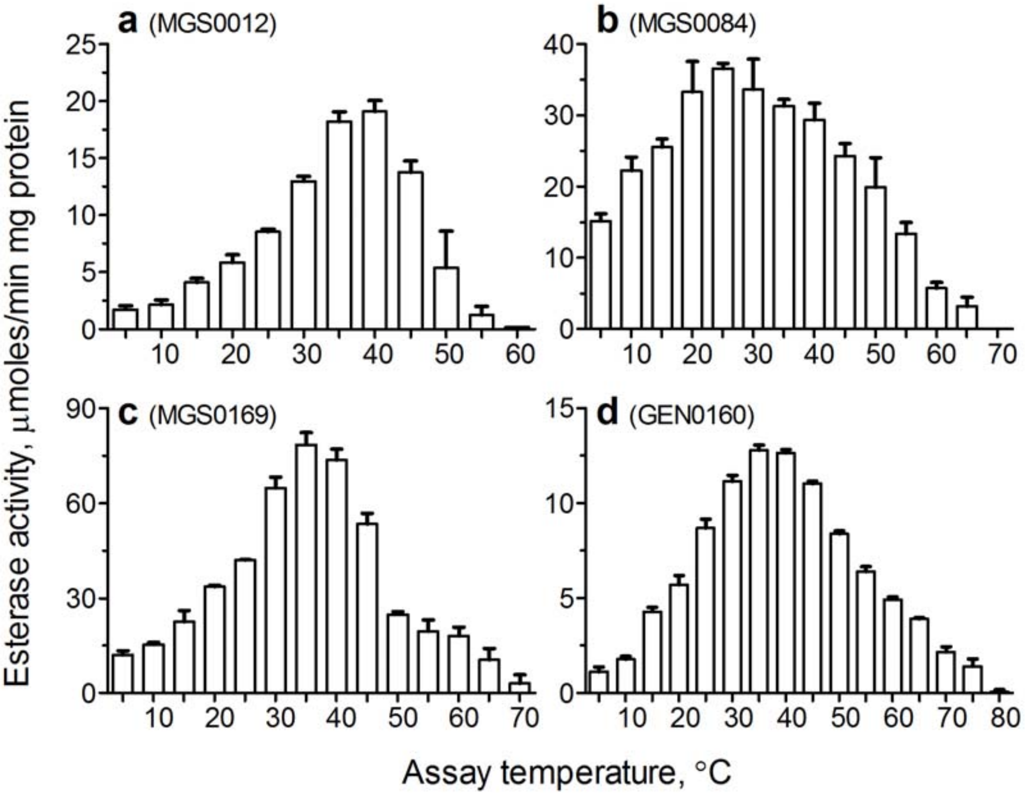

Supplementary Figure S5

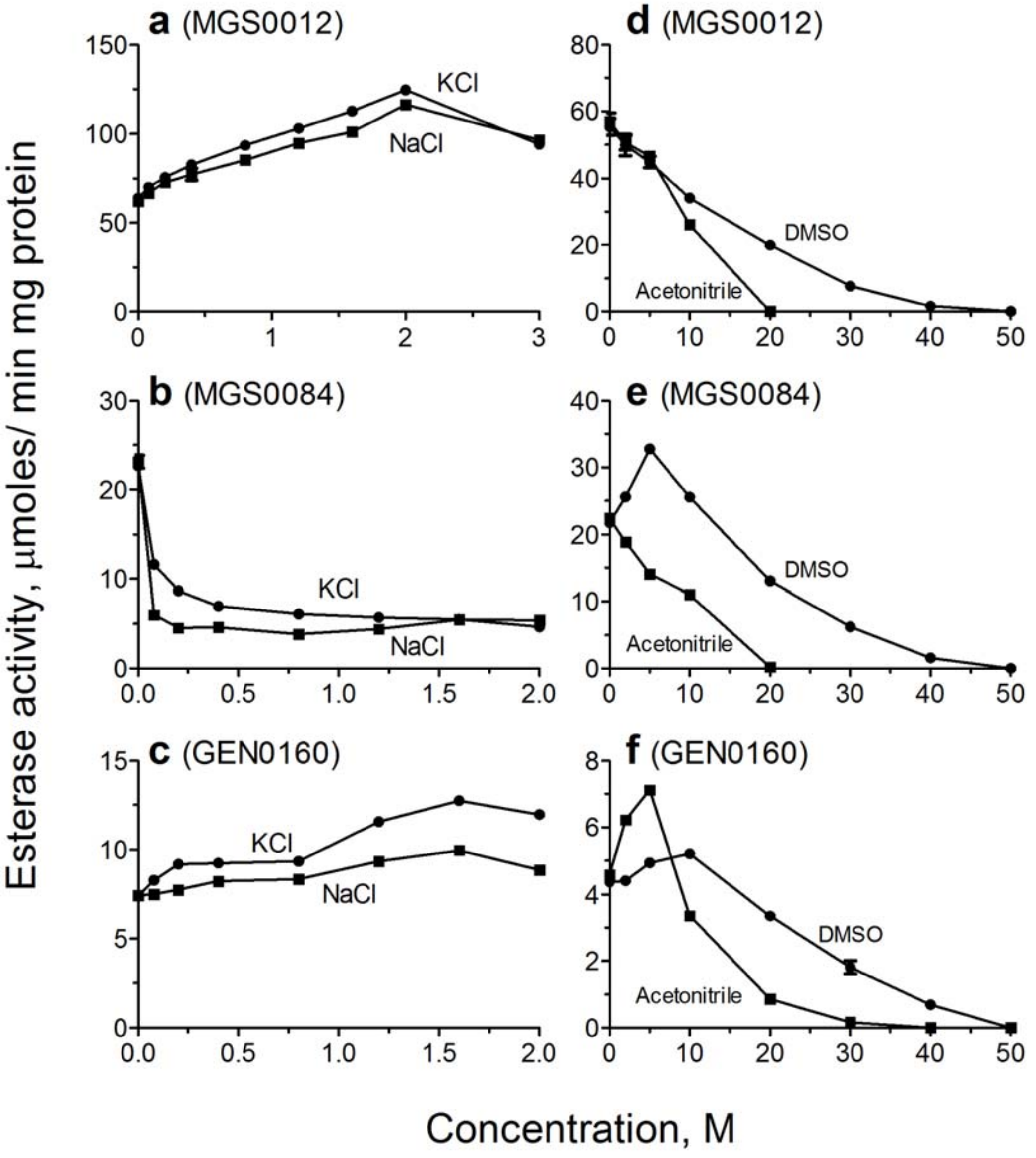

Supplementary Table S1. 16 metagenomic libraries used in this work.

Metagenomic library and location	Environmental conditions		Native sample or enrichment conditions
	Temperature	Salinity	
1. Anaerobic digester, waste water treatment plant (Genoscope, Evry, France)	33 °C	unknown	native sample
2. Composting plant, Liemehna (Germany)	30 - 50 °C	unknown	native sample
3. Kolguev Island, oil-contaminated coastal water, Barents Sea (Russia)	3 °C	3.1% – 3.8%	crude oil enrichment, 4 ºC
4. Messina harbour, Mediterranean Sea (Italy)	15 °C	3.1% – 3.8%	phenanthrene + pyrene (500 mg/L), 15 ºC
5. Messina harbour (Int II), Mediterranean Sea, (Italy)	15 °C	3.1% – 3.8%	naphthalene enrichment
6. Michle, PAH contaminated soil from wood processing industry (Czech Republic)	20 - 25 °C	unknown	native sample
7. Milazzo, Mediterranean Sea (Italy)	18 °C	3.1% – 3.8%	phenanthrene + pyrene (500 mg/L)
8. MT Haven sunken shipwreck, tar samples, Arenzano harbour, Ligurian Sea, Genoa (Italy)	13 - 14.4 °C	3.8 %	native sample
9. Priolo oil refinery, anoxic harbour sediment contaminated with crude and refined oil, Priolo Gargallo (Italy)	15 °C	3.1% – 3.8%	native sample
10. Port of Murmansk, oil-contaminated, Barents Sea (Russia)	5 °C	3.1% – 3.8%	crude oil enrichment, 4 ºC
11. *Rimicaris exoculata* gill, deep sea hydrothermal vent (Mid-Atlantic Ridge)	3 - 25 °C	3.3 – 3.7%	native sample
12. *Rimicaris exoculata* gut, deep sea hydrothermal vent (Mid-Atlantic Ridge)	3 - 25 °C	3.3 – 3.7%	native sample
13. Sobeslav soil, PAH contaminated bioremediation site (Czech Republic)	unknown	unknown	native sample
14. Tembec Paper Mill, Temiscaming (Ontario, Canada)	25 - 35 °C	unknown	native sample
15. Urania, deep hypersaline anoxic basin interface (Mediterranean Sea)	14 °C	ca. 15%	oil enrichment
16. Vulcano acidic pool, Vulcano Island, Mediterranean Sea (Italy)	ambient	unknown	heterotrophic enrichment

Supplementary Table S2. GenBank accession numbers and family classification of 80 metagenomic esterases.

Clone	(Meta)genomic Library	GenBank ID	COG	Superfamily (Clan)	Pfam
1. ABO0195	Murmansk	WP_011587492	COG0657	CL0028	PF07859.10
2. CC0144	*C.* sp. 78-ME	WP_015006593	COG0596	CL0028	PF12697.4
3. CC0149	*C.* sp. 78-ME	WP_016390780	COG0657	CL0028	PF07859.10
4. CC0150	*C.* sp. 78-ME	WP_015005403	COG2755	CL0264	PF13472.3
5. CC0151	*C.* sp. 78-ME	WP_020161852	COG0596	CL0028	PF00561.17
6. GEN0031	Genoscope	KP347712	COG1680	CL0013, CL0051	PF00144.21, PF13026.3
7. GEN0034	Genoscope	KP347713	--	CL0028	PF11288.5
8. GEN0042	Genoscope	KP347714	COG1680	CL0013	PF00144.21
9. GEN0071	Genoscope	KP347716	COG1752	CL0323	PF01734.19
10. GEN0083	Genoscope	KP347717	COG0627; COG2382	CL0028; CL0369	PF00756.17; PF02922.15
11. GEN0086	Genoscope	KP347718	COG0627	CL0028	PF00756.17
12. GEN0093	Genoscope	KP347719	COG0657	CL0028	PF07859.10
13. GEN0104	Genoscope	KP347720	COG1752	CL0323	PF01734.19
14. GEN0105	Genoscope	KP347721	COG0657	CL0028	PF07859.10
15. GEN0108	Genoscope	KP347722	COG1752	CL0323	PF01734.19
16. GEN0109	Genoscope	KP347723	COG0627; COG2382	CL0028; CL0369	PF00756.17; PF02922.15
17. GEN0111	Genoscope	KP347724	COG0596	CL0028	PF12697.4
18. GEN0112	Genoscope	KP347725	COG0627	CL0028	PF00756.17
19. GEN0115	Genoscope	KP347726	COG1075	CL0028	PF05057.11
20. GEN0135	Genoscope	KP347727	COG0596	CL0028	PF00561.17; PF12697.4
21. GEN0139	Genoscope	KP347728	COG0657	CL0028	PF07859.10
22. GEN0143	Genoscope	KR919778	COG0596	CL0028	PF00561.17
23. GEN0144	Genoscope	KP347729	COG2755	CL0264	PF13472.3
24. GEN0159	Genoscope	KP347730	COG0596	CL0028	PF12697.4
25. GEN0160	Genoscope	KP347731	COG0596	CL0028	PF12146.5
26. GEN0162	Genoscope	KP347732	COG0596	CL0028	PF12697.4
27. GEN0165	Genoscope	KP347733	COG0596	CL0028; CL0296; CL0063	PF00561.17; PF16884.2; PF00107.23
28. GEN0169	Genoscope	KP347734	COG0596; COG4099	CL0028; CL0381	PF10503.6; PF00753.24
29. GEN0170	Genoscope	KP347735	COG1680	CL0013	PF00144.21
30. GEN0175	Genoscope	KP347736	COG0596; COG1075	CL0028	PF12146.5
31. GEN0176	Genoscope	KP347737	COG1647	CL0028	PF12146.5
32. GEN0179	Genoscope	KR919777	COG3240	CL0264	PF00657.19
33. GEN0183	Genoscope	KP347738	COG1680	CL0013	PF00144.21

34. Koil14	Kolguev	KP347739	COG0596	CL0028	PF00561.17
35. MGS0004	Messina	KP347740	COG0657	CL0028	PF07859.10
36. MGS0005	Messina	KP347741	COG0657	CL0028	PF07859.10
37. MGS0006	Messina	KP347742	COG0657	CL0028	PF07859.10
38. MGS0008	Messina	KP347743	COG0657	CL0028	PF07859.10
39. MGS0009	Messina	KP347744	COG0596	CL0028	PF00561.17
40. MGS0010	Messina	AHG30919	COG1680	CL0013	PF00144.21
41. MGS0012	Messina	KP347745	--	CL0028	PF11288.5
42. MGS0016	Milazzo	KP347746	COG2021	CL0028	PF00561.17
43. MGS0017	Milazzo	KP347747	COG0657	CL0028	PF07859.10
44. MGS0018	*R. exoculata* gill chamber	AGT96416	COG0657	CL0028	PF07859.10
45. MGS0019	Sobeslav	KP347748	COG2267; COG1647	CL0028	PF12146.5
46. MGS0032	Messina	KP347749	COG1073	CL0028	--; PF12262.5
47. MGS0076	Compost	KP347750	COG1680	CL0013	PF00144.21; PF11954.5
48. MGS0077	Compost	KP347751	COG0657	CL0028	PF07859.10
49. MGS0078	Compost	KP347752	COG0657	CL0028	PF00326.18
50. MGS0080	Compost	KP347753	COG4188	CL0028	PF12740.4
51. MGS0081	Haven	KP347754	COG0657	CL0028	PF07859.10
52. MGS0084	Haven	KP347755	--	--	--
53. MGS0085	Haven	KP347756	COG1680	CL0013	PF00144.21
54. MGS0086	Haven	KP347757	COG2272	CL0028	PF00135.25
55. MGS0087	Haven	KP688389	COG1680	CL0013	PF00144.21
56. MGS0089	Haven	KP347758	COG0657	CL0028	PF07859.10
57. MGS0090	Haven	KP347759	COG0657	CL0028	PF07859.10
58. MGS0091	Haven	KP347760	COG0657	CL0028	PF07859.10
59. MGS0092	Haven	KP347761	COG0657	CL0028	PF07859.10
60. MGS0093	Milazzo	KP347762	--	CL0028	PF00326.18
61. MGS0094	Priolo	KP347763	COG0657	CL0028	PF07859.10
62. MGS0095	Priolo	KP347764	COG0657	CL0028	PF07859.10
63. MGS0099	*R. exoculata* gut	KP347765	COG0596	CL0028	PF00561.17
64. MGS0101	*R. exoculata* gut	KP347766	COG1752	CL0323	PF01734.19
65. MGS0103	Sobeslav	KP347767	COG0657	CL0028	PF07859.10
66. MGS0105	Kolguev	AGT96414	COG1680	CL0013	PF00144.21
67. MGS0106	Kolguev	KP347768	COG0657	CL0028	PF07859.10
68. MGS0109	Kolguev	AGT96415	COG0596	CL0028	PF12697.4
69. MGS0153	Murmansk	WP_011588480	COG0596	CL0028	PF00561.17
70. MGS0156	PaperMill	KP347769	--	CL0028	--
71. MGS0157	Murmansk	KP347770	COG0657	CL0028	PF07859.10
72. MGS0164	Vulcano	KP347771	COG1505	CL0028; CL0186	PF00326.18; PF02897.12
73. MGS0165	Compost	KP347772	--	--	PF10605.6
74. MGS0168	Haven	KP347773	COG0657	CL0028	PF07859.10
75. MGS0169	Haven	KP347774	--	--	PF04199.10

76. MGS0171	*R. exoculata* gill chamber	KP347775	COG0657	CL0028	PF07859.10
77. MGS0204	Michle	KP347776	COG1752	CL0323	PF01734.19
78. MGS0205	Michle	KP347777	COG1680	CL0013	PF00144.21
79. MGS0216	Michle	KP347778	COG0596	CL0028	PF12697.4
80. Uroil41	Urania	ABM19244	COG0657	CL0028	PF07859.10

Supplementary Table S3. 89 ester substrates used for screening of purified metagenomic esterases[a].

1. 2-Bromoethyl acetate	31. Ethyl octanoate	61. Methyl α-bromophenylacetate
2. (-)-Ethyl-L-Lactate	32. Ethyl propionate	62. Methyl trans-cinnamate
3. (-)-Methyl (S)-2-chloropropionate	33. Ethyl propionylacetate	63. Methyl-(R) -(+)-3-bromo-2-methylpropionate
4. (+)-Methyl (R)-2-chloropropionate	34. Ethyl trans-cinnamate	64. Methyl(R)-(-)-3-hydroxybutyrate
5. (-)-Methyl-L-lactate	35. Ethyl tribromoacetate	65. Methyl-(R)-(-)mandelate
6. (+)-Methyl-D-lactate	36. Ethyl trifluroacetate	66. Methyl(S)-(+)-3-hydroxy-2-methylpropionate
7. (1S)-(+)-Dimenthyl succinate	37. Ethyl((4-ethylthio)benzoyl)amino) acetate	67. Methyl(S)-(+)-3-hydroxybutyrate
8. (1R)-(-)-Dimenthyl succinate	38. Ethyl-(S)-(-)-4-chloro-3-hydroxybutyrate	68. Methyl(S)-(+)-mandelate
9. (1S)-(+)-Menthyl acetate	39. Ethyl-2-ethylacetoacetate	69. Methyl-2-chloro-3-hydroxypropionate
10. (1R)-(−)-Menthyl acetate	40. Ethyl-3-bromo-2(bromoethyl)propionate	70. Methyl-4-(hydroxymethyl) benzoate
11. (1S)-(+)-Neomenthyl acetate	41. Ethyl-3-bromopropionate	71. Phenethyl cinnamate
12. (1R)-(−)-Neomenthyl acetate	42. Ethyl 4-hydroxy-3-methoxycinnamate	72. Phenyl acetate
13. (S)-(+)-Glycidyl butyrate	43. Ethyl-3-oxohexanoate	73. Propyl acetate
14. (R)-(−)-Glycidyl butyrate	44. Ethyl-a-bromoisobutyrate	74. tert-Butyl 3-hydroxypropionate
15. Benzyl (S)-(+)-Mandelate	45. Isobutyl acetate	75. Triacetin
16. Benzyl (R)-(-)-Mandelate	46. Isobutyl cinnamate	76. Tributyrin
17. Butyl acetate	47. Isopropenyl acetate	77. Tricaprin (C10:0)
18. Ethyl (R)-(−)-3-hydroxybutyrate	48. Methyl (R)-(+)-3-bromo-2-methylpropionate	78. Tricaproin (C6:0)
19. Ethyl -2 bromopropionate	49. Methyl (S)-(+)-3-hydroxy-2-methylpropionate	79. Tricaprylin (C 8:0)
20. Ethyl 2-chloropropionate	50. Methyl 2,2-dimethyl-3-hydroxypropionate	80. Tripropionin
21. Ethyl 4-bromobutyrate	51. Methyl 2-chloropropionate	81. Vinyl acetate
22. Ethyl 4-chloroacetoacetate	52. Methyl 2-hydroxyisobutyrate	82. Vinyl benzoate
23. Ethyl acetoacetate	53. Methyl 3-bromopropionate	83. Vinyl butyrate
24. Ethyl bromoacetate	54. methyl benzoate	84. Vinyl cinnamate
25. Ethyl caprate	55. Methyl bromoacetate	85. Vinyl decanoate
26. Ethyl chloroacetate	56. Methyl butyrate	86. Vinyl laurate
27. Ethyl fluoroacetate	57. Methyl chloroacetate	87. Vinyl methacrylate
28. Ethyl iodoacetate	58. Methyl cinnamate	88. Vinyl pivalate
29. Ethyl 2-methylacetoacetate	59. Methyl glycolate	89. Vinyl propionate
30. Ethyl octanoate	60. Methyl pyruvate	

[a] All substrates are available from Sigma.

Supplementary Table S4. Crystallographic data collection and model refinement statistics.

**Protein name**	**MGS0169**
PDB id	5IBZ
space group	$P2_12_12_1$
unit cell (Å)	a =64.5, b = 120.6, c = 148.8
wavelength (Å)	0.9794
resolution (Å)	35.37-1.61
number of unique reflections	150117
average redundancy	5.9/4.9
$R_{merge}{}^{a}$ (%)	0.083(0.48)
completeness (%)	99.9(98.5)
I/σ	18.8(2.88)
**Refinement statistics**	
R_{cryst} (%)	13.28
R_{free} (%)	16.14
Protein residues/solvent	1364/1657
**Rmsd from target values**	
bond lengths (Å)	0.09
bond angles (deg)	1.33
**Average *B* factors (Å^2)**	
protein whole chains	15.5
solvent	29.0/37.5
**Ramachandran Plot ^{b} (%)**	97/3.0/-

$^{a}R_{merge}=\Sigma_{hkl}\Sigma_i|Ii_(hkl_)-\langle I_{hkl}\rangle|/\Sigma_{hkl}\Sigma_i I_{i(hkl)}$, where $Ii_(hkl_)$ is the *i*th observation of reflection *hkl*, and $\langle I_{hkl}\rangle$ is the weighted average intensity for all observations *i* of reflection *hkl*. Numbers in parentheses are values for the highest-resolution bin.
$^{\mathbf{b}}$Ramachandran plot statistics favored/allowed/outlier.
